# Neural correlates underlying local and global processing during visual search across adulthood

**DOI:** 10.1371/journal.pone.0303796

**Published:** 2024-06-21

**Authors:** Gaelle Doucet, Jordanna A. Kruse, Noah Hamlin, Carole Peyrin, Nicolas Poirel

**Affiliations:** 1 Institute for Human Neuroscience, Boys Town National Research Hospital, Omaha, Nebraska, United States of America; 2 Department of Pharmacology and Neuroscience, Creighton University School of Medicine, Omaha, Nebraska, United States of America; 3 French National Centre for Scientific Research, Laboratoire de Psychologie et NeuroCognition, Universite Grenoble Alpes, Universite Savoie Mont Blanc, Grenoble, France; 4 LaPsyDÉ, French National Centre for Scientific Research, Université de Paris, Paris, France; 5 GIP Cyceron, Caen, France; 6 Institut Universitaire de France, Paris, France; Bournemouth University, UNITED KINGDOM

## Abstract

Visual processing relies on the identification of both local and global features of visual stimuli. While well investigated at the behavioral level, the underlying brain mechanisms are less clear, especially in the context of aging. Using fMRI, we aimed to investigate the neural correlates underlying local and global processing in early and late adulthood. We recruited 77 healthy adults aged 19–77 who completed a visual search task based on 2-level hierarchical stimuli made of squares and/or circles. Participants were instructed to detect a target (a square) at either a local (small) or global (large) level of a hierarchical geometrical form, in the presence or absence of other hierarchical geometrical forms (distractors). At the behavioral level, we revealed high accuracy for all participants, but older participants were slower to detect local targets, specifically in presence of distractors. At the brain level, while both local and global processing were associated with occipital activation, local processing also recruited the anterior insula and dorsal anterior cingulate cortex, that are core regions of the salience network. However, while the presence of distractors in the local condition elicited specifically stronger activation within the right anterior insula for the young group, it was not observed for older participants. In addition, older participants showed less activation than younger participants in the occipital cortex, especially for the most complex conditions. Our findings suggest that the brain correlates underlying local and global processing change with aging, especially for complex visual patterns. These results are discussed in terms of top-down reduction effects from the salience network on primary visual areas, that may lead to specific difficulties to process local visual details in older adults.

## Introduction

Visual processing largely relies on the identification of the hierarchical levels of visual stimuli, from the largest global level to the most local elements. The overall challenge in studying visual processing comes from the fact that in a real-world situation, the global level can be predicted from the identity of the local level and vice versa. One solution to investigate these complementary aspects of visual processing has been to use a task that includes compound forms as stimuli [1], consisting of large letters (the global level) composed of a suitable arrangement of several small letters (the local level). The global letter can be identical (congruent) or not (incongruent) to the local letters. In such paradigms, participants are for example instructed to focus on a level (e.g., global) and decide whether a target letter was present, while ignoring the other ‘‘irrelevant” unattended level (e.g., local). Such visual tasks led to the reproducible and robust finding that people are faster at detecting information at the global, relative to the local level [1, 2], which has been referred to as the global precedence effect (GPE). In addition, incongruent global information usually slows down the detection at the local level, but incongruent local information has very little effect on the detection at the global [1, 2], which has been referred to as the global interference effect.

Since this original work by Navon [1], it has been suggested that the GPE is related to different levels of attentional resources allocated to either level, such as global level gets larger allocation than local level, leading to more easily and efficiently processing for the former [3]. However, while largely replicated in youth [4] and young adults [2], studies have reported mixed results in older populations. For instance, some studies have described changes from global to local bias with healthy aging [5–8], indicating better local processing in older, compared to younger adults. In contrast, others have found either no or a facilitating impact of aging on the global precedence effect [9–14]. Such discrepancies may be related to variation in tasks and stimuli’s characteristics [15] or differences in brain mechanisms such as age-related alteration in inhibitory mechanisms that are thought to support the interference effect [2].

From studies done on young adults, the degree of interference effect from salient global information can also be influenced by the number of visual items presented at once (e.g., addition of visual distractors), as it adds up another degree of interference [16, 17]. For example, Krakowski et al. [17] used a visual search task in which a target (a geometrical form) could appear at one of three levels of a hierarchical stimulus. Critically, the hierarchical stimulus containing the target was displayed among a varying number of other hierarchical stimuli called distractors. By varying the number of distractors, the detection of targets presented at a global level remain very efficient (i.e., the detection times did not vary with the number of distractors) whereas local targets were processed less and less efficiently (i.e., the detection times increased with the number of distractors). In this context, executive function has been suggested to be playing a critical role in such visual search tasks, especially in regard to ignoring distractors to be able to correctly select a target [17–20]. Interestingly enough, using a similar visual search task, Bouhassoun et al. [10] revealed that the interference effect of distractors on the detection of local, but not global, targets was more pronounced in older adults (> 50 years old) than in younger adults, which may suggest aging-related alteration in top-down and inhibitory mechanisms and/or a more limited capacity for processing local information in older adults. Alternatively, this could be related to age-related alteration in early sensory areas with increasing processing demands [21]. However, to our knowledge, the neuronal mechanisms underlying local/global processing during a visual search task across adulthood have not yet been investigated, which prevents inferences on the biological origins of such age-related alterations.

In early adulthood, the pioneer neuroimaging studies that used hierarchically organized visual stimuli on detecting local versus global information were actually conducted to investigate functional brain asymmetries involved in the processing of global and local elements [22–25]. For this purpose, they presented single compound letter forms. More recently, with the development of more complex tasks such as visual search tasks, functional MRI (fMRI) studies have confirmed the recruitment of brain networks typically supporting executive functions, such as the central-executive and salience networks [26–29]. The central executive network, covering lateral fronto-parietal cortices, is typically recruited during working memory and executive functions as well as inhibitory mechanisms [18, 30, 31]; while the salience network, largely including anterior insula and dorsal anterior cingulate cortex (dACC), has been involved in the identification of biologically and cognitively relevant events to guide flexible behavior, including the detection and selection of salient stimuli [32–34]. Overall, these two networks have been suggested to evaluate the salience status of the visual stimuli and help process the targets in a competitive visual environment required by the task demands, respectively. In particular, the salience network has been reported during the visual processing of incongruent (i.e., local and global levels of a stimulus do not match) versus congruent (i.e., local and global levels of a stimulus match) visual stimuli, as well as during local visual processing [26, 35–37]. In contrast, global processing has been rather associated with the activation of the central-executive network [26]. However, it should be noted that both networks have also been typically detected as activated during more traditional visual search fMRI tasks that did not involve any local or global level of detection [29, 38, 39]. In this context, these networks have been suggested to support all stages of visual search with each network supporting more specific mechanisms. Overall, these findings have been more largely investigated in early adulthood and it is unclear which underlying neural network may be more impacted by aging during visual search tasks and lead to behavioral differences between early and late adulthood as described above. An fMRI study [29] conducted on healthy adults aged 18–78 years suggested age-related differences in activation in the fronto-parietal network during a visual search task, which they linked to overall efficiency of search and the saliency of the visual targets. While this task involved different types of distractors, it did not involve any local or global targets.

**Fig 1:**
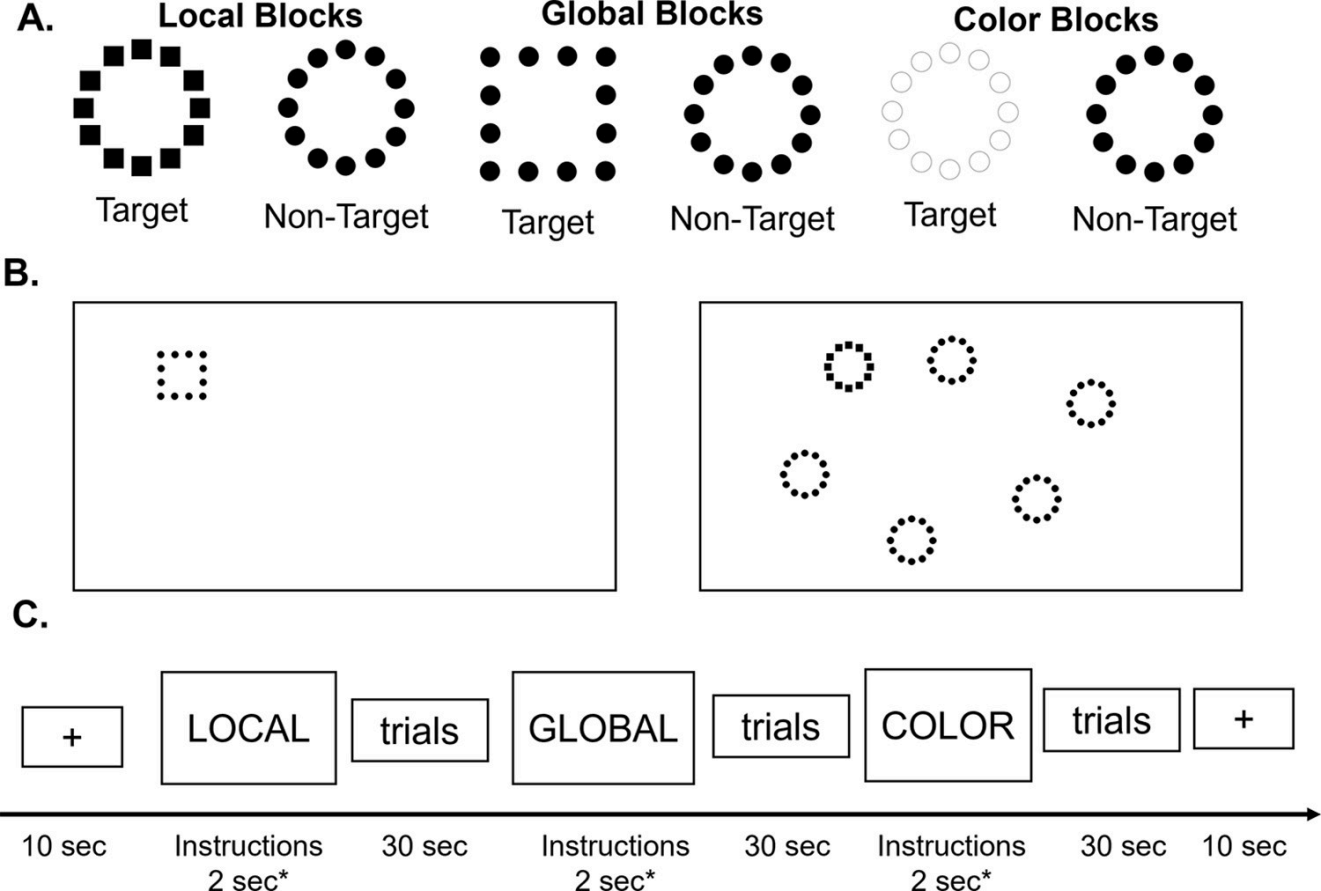
Design of the fMRI task. (A) Hierarchical stimuli with two levels (local and global). Example of target and non-target stimuli for each condition. (B) Example of target-present Global-0D (left panel), and Local-5D (right panel) trials. (C) Simplified design of one fMRI scan. Each block lasted approximately 30 sec. The order of the blocks was pseudo-randomized between subjects and between runs, with a total of 10 blocks within a run. Each functional scan started and ended with a 10-sec rest.

In this context, we aimed to investigate the brain mechanisms behind local and global processing during a visual search task of compound hierarchical stimuli in early and late adulthood. Healthy younger and older adult individuals were recruited and completed a fMRI visual search task. This task was an adaptation of a behavioral paradigm published previously [10, 17], which has been simplified to be fMRI compatible. The task was based on two-level hierarchical stimuli that were a global geometrical form (a circle of a square) composed of several local geometrical forms (circles or squares; Fig 1). Participants were instructed to detect a target geometrical form (i.e., a square) at either the local or global level. In order to investigate the impact of visual distractors during global and local processes (and the related top-down mechanisms), the hierarchical target could be presented either alone or with five other hierarchical forms called distractors (Fig 1B).

Due to well-known interference of global information during the local processing, we first hypothesized that the salience network (i.e., dACC and anterior insula) would be more largely recruited during the local, compared to the global conditions across all participants [10, 26]. More important with respect to our research objectives, we expected that the presence of distractors in the local condition increased top-down processing to inhibit non-target stimuli. However, our previous behavioral results showed that older participants were less efficient than younger participants for processing local information in particular in the context of increased visual competition (i.e. when multiple visual stimuli were displayed; [10]), suggesting altered top-down processing with healthy aging. Therefore, in the present study, we hypothesized that in the local condition only, the younger group will show a greater activation of the salience regions in the five-distractor condition than the no-distractor condition, in contrast to the older adults who will show less activation of the salience regions.

## Materials and methods

### Participants

A total of 77 healthy adult participants were recruited and divided into two age groups: one young group of 39 participants aged 20–33 years-old and one older group of 38 participants aged 51–77 years-old. All participants were free of psychiatric or neurological disorders. Participants requiring visual correction wore the Adult MediGoggle Set (MRIMed; https://www.mrimed.com/Item/pg-101), interchangeable prescriptive goggles suitable for use in MR environments. Four participants were excluded from further analyses because of low accuracy during the visual search task (<80%, n = 2), severe head motion during the MRI acquisition (n = 1) or poor vision that could not be adjusted with the MRI-compatible glasses (n = 1); which resulted in a total of 38 younger adults (mean age ± sd = 26.13 ± 3.50 years, 21 women) and 35 older adults (mean age ± sd = 62.16 ± 6.70 years, 22 women). Groups did not significantly differ in gender, handedness, or education level (*p’s*>0.6). Despite having no participants with a Mini-Mental State Examination (MMSE) score under 25, the younger group had a very slightly, but significantly higher MMSE score than the older group (younger: mean = 29.4±0.7; older: mean = 28.8±1.2, t = 2.6, *p* = 0.01). The study was approved by the Institutional Review Board for Research with Human Subjects at Boys Town National Research Hospital. All participants provided a written informed consent.

### MRI data acquisition

Participants were scanned on a 3T Siemens Prisma scanner using a 32-channel head coil. Structural images were acquired using a T1-weighted, 3D magnetization-prepared rapid gradient-echo (MPRAGE) sequence with the following parameters: Repetition Time (TR) = 2400 ms, Echo Time (TE) = 2.05 ms, Field of View (FOV): 256×256 mm, 1 mm isotropic resolution, Inversion Time (TI) = 1000 ms, 8 degree-flip angle, bandwidth = 240 Hz/Pixel, echo spacing = 7.0 ms, in-plane acceleration GRAPPA (GeneRalized Autocalibrating Partial Parallel Acquisition) factor 2, total acquisition time ~6 min. Participants completed two functional scans of a visual search task, using a multi-band T2* sequence with the following acquisition parameters: TR = 480 ms, TE = 29.2 ms, voxel size = 3x3x3 mm^3^, 44 degree-flip angle, echo spacing = 0.51 ms, bandwidth = 2772 Hz/Pixel, number of axial slices = 56, multi-band acceleration factor = 8, duration = 4min9sec. For each functional scan, 725 volumes were collected. Stimuli were displayed using E-prime 3 software. They were displayed onto a screen positioned at the rear of the MRI magnet, duplicating the main computer screen in the console room. Participants viewed the screen through a mirror fixed on the head coil.

### Visual search task

Stimuli were hierarchical forms made of circles and/or squares either all black or all white with a black outline (Fig 1A). Target hierarchical forms were either a global circle made of small local squares (local condition), or a large global square made of small local circles (global condition). Non-target and distracting hierarchical forms were always a global circle made of local circles. The target hierarchical form could be presented either alone or with five other hierarchical forms called distractors (Fig 1B) in order to investigate the impact of visual distractors during global or local processing.

Using a block design, the task included three main conditions: (1) a condition called LOCAL, during which participants were instructed to determine whether “small” squares were present or not among the hierarchical stimuli displayed on the screen; (2) a condition called GLOBAL, during which participants were instructed to determine whether a “large” square was present or not among the hierarchical stimuli displayed on the screen; and (3) a control condition called COLOR, during which participants were instructed to determine the color of the stimuli on the screen (white or black) (Fig 1A). This condition was used to control for attentional processing and ensure that participants were able to see the stimuli regardless of their composition. The stimuli’s positions on the screen were balanced across all conditions. In the conditions LOCAL and GLOBAL, participants responded by pressing their index finger for “square present” (at their respective level), and their middle finger for “square absent” (*i*.*e*., only circles at both local and global levels) as quickly and as accurately as possible. In the condition COLOR, all stimuli were composed of circles (at both local and global levels) and were all the same color (either white or black); participants pressed their index finger for white stimuli and their middle finger for black stimuli.

Overall, the task included six conditions: Local 0 Distractor (L-0D), Local 5 Distractors (L-5D), Global 0 Distractor (G-0D), Global 5 Distractors (G-5D), Color 1 Stimulus (C-1S), Color 6 Stimuli (C-6S, all the same color). These conditions in terms of number of distractors (5D vs. 0D) and the levels of target detection (local vs. global) were specifically chosen based on our previous study that demonstrated that these conditions showed the strongest effect with the age groups [10]. We therefore expected that the comparisons across these conditions would show the strongest difference in brain activation between the younger and older adults.

The experiment included two functional scans, each composed of 10 blocks: two blocks for the L-0D, L-5D, G-0D and G-5D conditions, and one block for the control C-1S and C-6S conditions. The order of blocks was pseudorandomized within a scan and also between participants. A simplified example of the timing of a functional scan is shown in Fig 1C. A functional scan started and ended with a 10 sec rest period. Instructions appeared at the start of each block for 2 seconds. A block of trials lasted 30 seconds and participants responded to as many stimuli as they could within this 30-second period. Thus, each trial started with the presentation of a fixation cross for 500 ms, and followed by the stimuli that remained on the screen until the participant provided a response. This was chosen over a specific and fixed time for each stimulus display to ensure the ability to measure GPE in each individual, in a block design. We also ensured that a target was present in approximately half of the trials in each block and no more than three trials in a row.

A training session was conducted for each participant before the fMRI task to ensure that they could accurately see the stimuli, and correctly perform the task. Response times (RTs) and accuracy were recorded. Participants were instructed to respond as quickly as possible while still being correct.

### FMRI preprocessing

The fMRI data were preprocessed using Statistical Parametric Mapping (SPM12) and the DPABI Toolbox [40]. For both runs, preprocessing procedures included motion correction to the first volume with rigid-body alignment, co-registration between the functional scans and the anatomical T1-weighted scan, spatial normalization of the functional images into Montreal Neurological Institute (MNI) stereotaxic standard space, and spatial smoothing within the functional mask with a 6-mm at full-width at half-maximum Gaussian kernel.

### FMRI task whole brain activation

General linear model analyses were implemented using SPM12. First, the preprocessed single-participant images from both runs were combined. At the first level, six conditions of interest (L-0D, L-5D, G-0D, G-5D, C-1S, and C-6S) were modeled as six regressors constructed as box-bar functions convolved with a canonical hemodynamic response function for each individual. We also included a regressor for the run order and six regressors for the head motion as factors of no-interest. At the individual level, we further identified the brain regions involved in the local and global visual processing, relative to the color control condition (L-0D > C-1S, L-5D > C-6S, G-0D > C-1S, G-5D > C-6S).

At the second level, whole-brain analyses were performed. An ANOVA and further post-hoc t tests were performed based on individual analyses by means of a full-factorial design. The ANOVA included the age group (young versus older) as a between-subject factor, while level (local versus global) and number of distractors (0 versus 5D) were considered as independent within-subject factors. Gender and total number of trials in each condition were added as covariates of no interest. We used the number of trials as a covariate in order to cancel potential effects of habituation for a group that would see more stimuli. Based on our hypotheses, we mainly focused on the interaction between age groups (young and older participants) and the levels of processing (global and local level) and pairwise comparison test if the interaction was significant. It should be noted that the color blocks were used as control for each level investigated. Therefore, when reporting the network supporting the local level processing, the contrast local > color was actually utilized, and so on for the level processing and sub-conditions (L-0D > CS-1S, G-0D > C-1S, and L-5D > C-6S, G-5D > C-6S).

For the main effect analyses, significant networks were identified using a whole brain threshold of *p*<0.05 (family wise error (FWE) corrected) and a minimum number of 5 voxels. For the post-hoc analyses, significant threshold was set up at *p*<0.001 (uncorrected, whole brain level) and *p*<0.05 (FWE corrected at the cluster level).

### Exploratory region-of-interest (ROI) analyses

Lastly, we conducted exploratory analyses using a ROI approach to specifically investigate the association between behavioral performance and brain activation. The number of recent fMRI studies investigating local and global visual processing using hierarchical stimuli in healthy adults has been relatively sparse and none have been using identical tasks. In this context, we chose one of the most recent studies done by Liddell et al. [26] that investigated the visual attention networks during global vs. local processing in 42 healthy young adults. Their task indexed perceptual conflict processes by manipulating attention towards the global (large shape) or local (small shape) level in composite hierarchical stimuli composed of different shapes, similar to our study. Using their study, we identified six ROIs located in the lateral parietal and frontal regions activated during both local > control and global > control contrasts and two ROIs located in the right anterior insula and anterior cingulate cortex activated during the local > control contrast (S1 Fig in S1 File). Activated regions located in sensory and cerebellar regions were not used.

For each of the eight ROIs, we extracted mean parameter estimates within 6-mm radius sphere centered on the peak of activation, for each major contrast, using the Marsbar toolbox [41]. Pearson’s correlation analyses were conducted to test the association between the ROIs’ mean BOLD signal and the in-scanner performance using efficiency scores [42, 43], for each of the four visual search conditions of interest (i.e., L0D, L5D, G0D, G5D), respectively. These performance scores are derived from a ratio between the percent accurate and mean RTs for accurate responses. We chose to use efficiency scores as they provide an integrative score of both RTs and accuracy rates and do not show ceiling effects such as those seen with the accuracy rates for this task [10]. They also have been previously validated [43] and used in other large studies such as the Adolescent Brain Cognition Development project [42, 44].

## Results

### Behavioral results

The overall accuracy was very high for both age groups across all conditions (young group (mean±sd): 95.0±1.9%; older group: 93.8±3.5%, *p* = 0.065). Across runs and participants, the average number of trials performed for the global condition in 4 blocks was 197 (sd = 28), for the local condition in 4 blocks was 148 (sd = 24), and for the color condition in 2 blocks was 109 (sd = 13), and was significantly higher for the younger than for the older group (p<0.001 for all). Consistently, a repeated-measure ANOVA conducted on the RTs to detect the target stimuli during the local and global blocks revealed a main effect of age groups (F = 125.3, *p* <0.001). Overall, younger adults were faster (672±81 ms) than older adults (969±140 ms) to detect the targets. There was also a significant main effect of the level of target (F = 441.1, *p* <0.001) and a significant group × level of target interaction (F = 23.9, *p* < 0.001). Both age groups were slower to detect the targets at the local than at the global level, confirming a global precedence effect described in previous behavioral studies (global: 565±71 ms and 800±126 ms; local: 869±119 ms and 1261±215 ms for younger and older participants, respectively). The group × level of target interaction revealed that older participants were disproportionately slower than younger participants during local processing compared to global processing.

**Fig 2:**
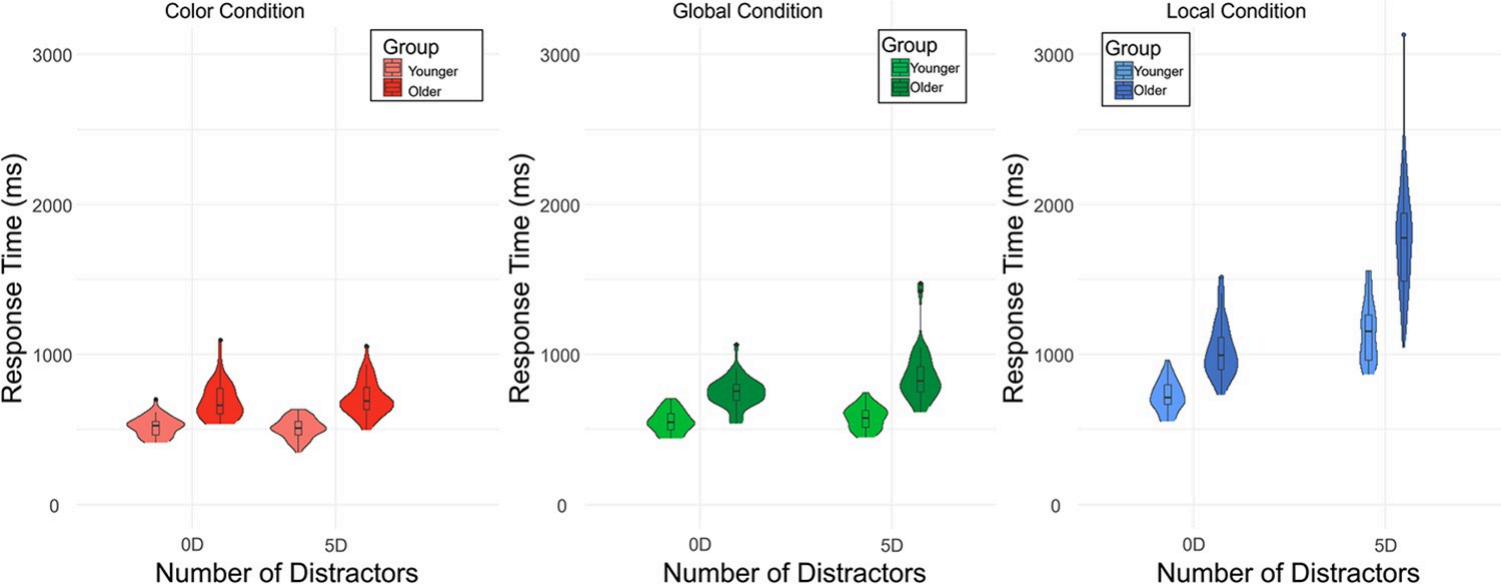
Response times in milliseconds by target level and number of distractors in present-target trials, in each age group.

Furthermore, we observed the expected group × level of target x number of distractors interaction (F = 15.3, *p* < .001; Fig 2). In detail, compared to younger participants, older participants were even more slower to detect local targets in the context of a larger number of distractors displayed.

Lastly, in the color (control) condition, we only revealed a significant main effect of age group (i.e., older slower than younger participants, F = 78.5, p < .001), but there was no main effect of number of stimuli (F = 0.06, *p* = 0.8) nor an interaction with the age group (F = 2.9, *p* = 0.09).

We also tested that the use of MRI-compatible glasses did not impact the behavioral results. There was no significant main effect of wearing glasses (i.e., participants wearing MR-compatible glasses vs. participants who did not) or any interactions with it (age x eyeglasses groups) for any of the accuracy or RT metrics for any conditions.

All results remain the same after removing an outlier in the older group (see Fig 2, participant with a mean RT > 3000 ms for the Local-5D condition).

### Whole-brain results

**Fig 3:**
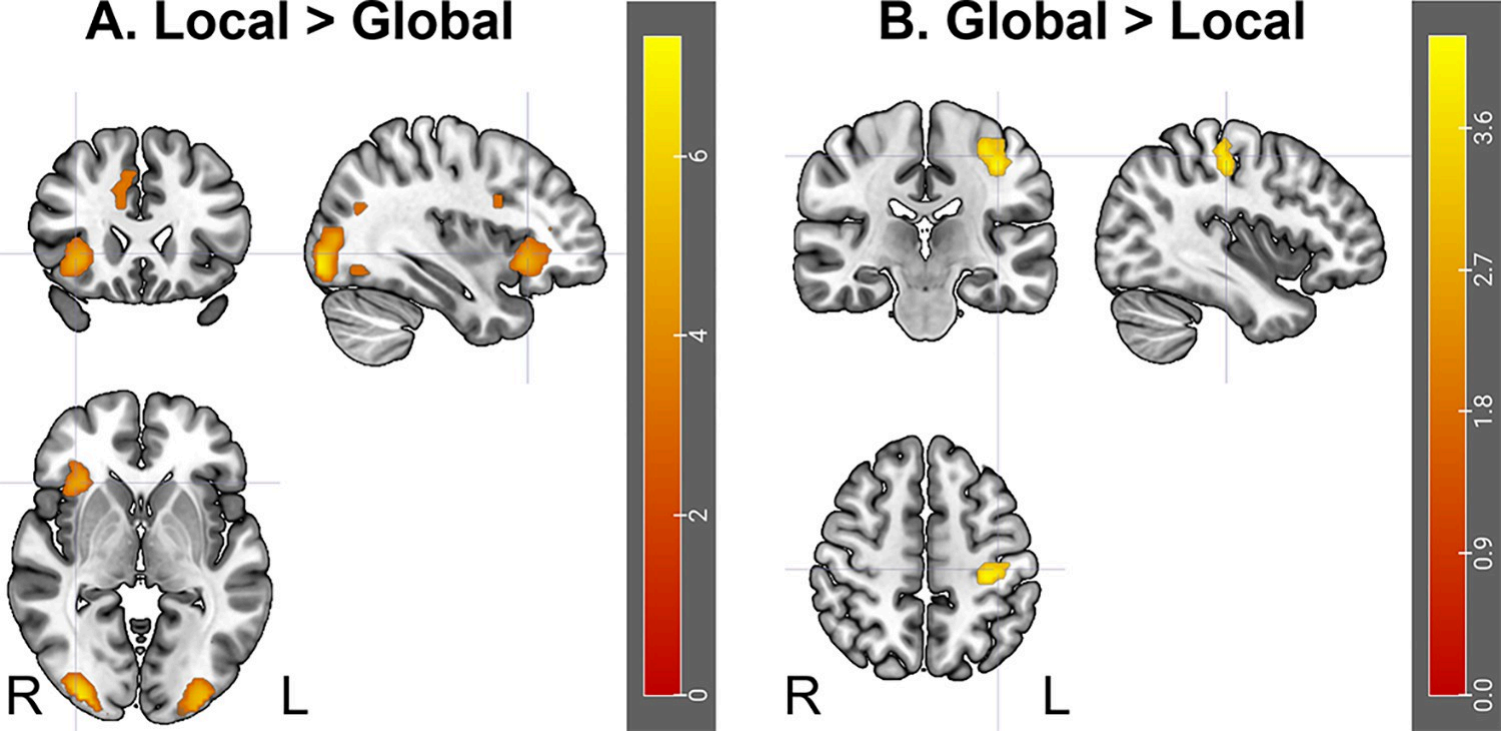
Activation underlying the local and global processing, across all participants. Activations are presented in a radiological view. R; Right, L: Left Hemisphere.

We first investigated the activation associated with each target level (local and global processing) across all participants. Global (relative to color) processing was associated with activation in a network largely covering the occipital lobe, including bilateral calcarine, cuneus, lingual gyrus, as well as middle, and superior occipital gyri, extending to both the medial parietal lobe and the posterior hippocampi, bilaterally. Local (relative to color) processing was associated with the same activation in the occipital lobe (largely covering bilateral calcarine, cuneus, lingual gyrus, as well as middle, and superior occipital gyri; detail in S1 Table in S1 File). However, this network also covered some additional frontal regions, including bilateral anterior insulas, middle frontal cortices and dorsal anterior cingulate cortex (dACC)/supplementary motor area (SMA). Consistently, the direct comparison of the two networks revealed higher activation in the inferior occipital cortices, right anterior insula, dACC/SMA, and right inferior frontal cortex in the local, relative to the global condition (S1 Table in S1 File and Fig 3). In contrast, global processing was associated with higher activity in the left precentral gyrus, compared to the local processing, which is likely related to behaviorally more motor responses in this condition.

In regard to the various interactions, we first found a significant triple interaction (i.e., age group-by-target level-by-distractor number interaction), with a significant cluster in the left pre/postcentral cortex (F = 15.84, k = 57, x = -36, y = -21, z = 51). Following this and based on our hypotheses, we further investigated several 2-by-2 interactions as described below. The interaction between the group (young and older participants) and the number of distractors did not reveal any significant clusters. In contrast, the interaction between the group and the level of processing (global and local) was associated with a significant activation of the cuneus (F = 18.96, k = 96 voxels, peak coordinates: x = -6, y = -72, z = 21). The nature of the interaction was explored firstly by comparing the processing of global and local levels for each group (within-group analysis), and then by directly comparing the groups for each level of processing (between-group analysis).

The within-group analysis revealed that the [Local > Global] contrast activated for each group the same network as the one observed for the whole group (i.e., lateral occipital cortices and right insula; Fig 3). The reverse [Global > Local] contrast was not significant. Based on our hypothesis, we further investigated the differences between the local and the global processing by also considering the number of distractors. Whatever the number of distractors (0 or 5) or the group (young or older), the same network was involved in the processing of the local level, in comparison to the global level. More interesting results were revealed by the direct comparison between 5 and 0 distractors ([0D > 5D] and [5D > 0D] contrasts) for each group and each level of processing. For the global level, there was a significant activation for the young group only: the condition with 5 distractors elicited stronger activation in a large occipital cluster covering the cuneus, lingual gyrus, and middle occipital cortex (k = 1580 voxels, T = 7.91, peak coordinates: x = -15, y = -84, z = -6) than the condition without distractors. For the local level, there was again a significant activation for the young group only: the condition with 5 distractors elicited stronger activation in the occipital cortex (cuneus, lingual gyrus, extending to the superior and middle occipital cortices) and right anterior insula than the condition without distractors (S1 Table in S1 File). Critically, there was no significant difference in activation when contrasting 5 to 0 distractors for the older group (whatever the level of processing, global or local). It should also be noted that there was no significant difference in activation when contrasting 0 to 5 distractors, likely due to less information to process when there was only one than six hierarchical stimuli.

**Fig 4:**
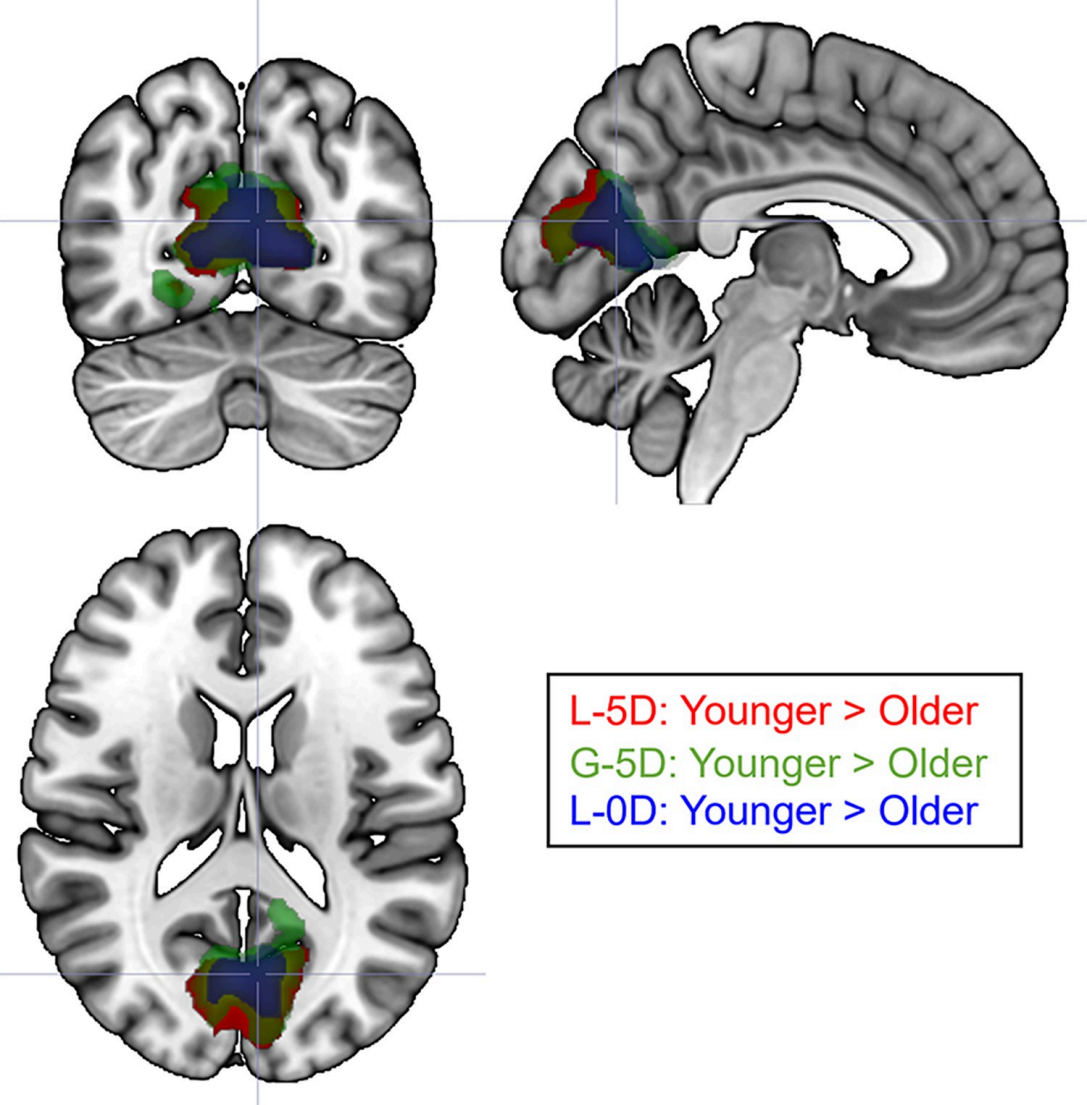
Between-group differences in occipital activation for the L-5D, G-5D and L-0D conditions. There was no significant difference for the G-0D condition between the two age groups. All activations overlapped and were greater for the L-5D for the young adults than for the older adults.

The between-group analysis revealed a greater activation of the cuneus for young than older participants ([Young > Old] contrast) for both levels. It should be noted that the cluster of activation was twice as large in the local (1209 voxels) than in the global (446 voxels) condition. The reverse contrast ([Old > Young] contrast) was not significant. We further investigated the differences between young and older participants by considering the number of distractors. The between-group analysis conducted on each single condition (G-5D, G-0D, L-5D, L-0D) revealed again greater activation of the cuneus for young than older participants for the G-5D, L-5D, and L-0D conditions (Fig 4). Nonetheless, there was no significant difference in activation between the two groups for the G-0D condition.

Results remained similar when we reconducted the fMRI analyses without an outlier from the older group based on the behavioral results (see Fig 2).

### Exploratory ROI results

Lastly, we explored the activation levels in specific *a priori* ROIs located in parietal and frontal regions based on Liddell et al. [26] (S1 Fig in S1 File) and how they correlated with behavioral (in-scanner) performance, in each condition. While some associations were statistically significant at p<0.05, none survived a multiple-comparison correction (S2 Table in S1 File).

## Discussion

This study investigated the neural correlates underlying local and global processing during a visual search task using hierarchical compound stimuli, across adulthood. At the behavioral level, all participants showed a high rate of accuracy and both older and young adults showed faster responses for global, relative to local conditions. We also did not find any difference in behavioral responses between those wearing MRI compatible eyeglasses versus those who did not wear any, which suggests that the ability to see accurately the targets at both levels was relatively similar across all participants. We were able to reproduce previous findings from our behavioral study showing that in comparison to young adults, older participants (a) were faster for global than local conditions, confirming a GPE across adulthood, (b) were, however, slower to detect local targets, and (c) were more disturbed by distractors during the local than the global visual search [10]. This confirms the robustness of the findings and the proper adaptation of the behavioral task to an MRI environment. As found in our previous study [10], it seems that healthy aging does not strongly impact GPE. Importantly, the absence of behavioral differences in regard to GPE between the two age groups is consistent with the absence of cortical differences between the two groups for the global condition in the absence of distractors ([Old > Young] contrast for the G-0D condition). This suggests that, in the context of a simple visual environment, the visual processing of global features remains relatively intact in late adulthood. Importantly, in such a context, a bottom-up selection—indicated by the selection being determined by the feature properties present in the environment, regardless of top-down control [45]—is involved. However, as soon as the environment becomes more visually complex and involves top-down processing to select the correct target (i.e., local processing in presence of visual distractors), brain and behavioral differences appear with late adulthood (see also [29]), as discussed further below.

At the brain level, and irrespective of groups, we revealed strong activation in the temporo-occipital cortex, for both global and local processing. In line with the study by Liddell et al. [26], local processing further involved the recruitment of anterior insula, inferior frontal cortex and dACC—which have been typically described as regions of the salience network [32]—relative to global processing. In contrast, global processing was just associated with higher activity in the motor cortex, which is likely due to the higher number of responses provided for the global conditions, compared to the local conditions. In order to address our research objectives, we compared groups for each level of processing (between-group analysis), and then we compared activation elicited by global and local conditions in young and older participants separately (within-group analysis). The between-group analysis revealed differences only in the occipital cortex. The differences were characterized by a decrease of activation in both local conditions (with or without distractors), as well as in the global condition with distractors. No significant differences were observed concerning the easier global condition (i.e., G-0D; as also suggested by the behavioral results). The within-group analysis revealed differences only when considering the manipulation of the number of distractors. While the presence of distractors in both global and local conditions elicited stronger activation in the occipital cortex for the young group, which is consistent with the addition of visual inputs, this activation was surprisingly not observed for older participants. Critically, the presence of distractors in the local condition elicited specifically stronger activation within the right anterior insula for the young group, which was also not observed for older participants. The anterior insula is a core brain region that anchors the salience network [32, 34]. Such extra recruitment for younger participants is consistent with the cognitive role of this network which is involved in the identification of biologically and cognitively relevant events to guide flexible behavior [32–34]. In particular, the anterior insula has been defined as a critical hub for detection and selection of salient stimuli [34]. In contrast, we did not reveal any greater activation in the anterior insula during the detection of global targets in presence of visual distractors, in comparison to no distractor. These results are in line with behavioral studies suggested that global targets are detected automatically in a pop-out way, bottom-up selection, whereas local targets are detected more effortfully with increasing numbers of visual distractors [3, 17, 46, 47] and involves top-down mechanisms [3]. These previous studies described that such differences between local and global detections were related to increased competition for attentional resources during the local processing only. The current study seems to be consistent as it also suggests that local -but not global- processing during a visual search may require increasing recruitment of the salience network to detect the most local elements of compound stimuli in presence of multiple visual distractors. Such extra recruitment may then lead to slower reaction times. However, it will be important to further validate this hypothesis with more appropriate statistical tests such as multiple-regression analyses. In contrast, we support the idea that the global processing, described as more efficient and effortless, may need less frontal recruitment due to a passive automatic attentional capture through a bottom-up selection of global targets [45].

Critically, the activation of the insula was not observed in older participants during the local processing, suggesting a salience network impairment with healthy aging. This finding is contrasting with the study by Merenstein et al. [29] as the authors described age-related increases in activation in the insula during a visual search task (conjunction (T/F target letters among rotated Ts and Fs) > feature (T/F target letters among Os) contrast). Their diffusion decision analyses further suggested a significant mediation role of the insula activation in predicting an age-related decrease in the rate of evidence accumulation during the conjunction search. While both studies highlight the involvement of the insula during visual search tasks, it also underscores how the task design (including visual stimuli choices) impacts the activation of the brain network supporting visual processing. Given that the two fMRI tasks have many experimental differences (e.g., stimuli feature choice, number of distractors, distractor types), it is currently unclear what unique aspect may be the cause and it will be therefore essential to further investigate each design in independent studies. However, such large differences are in line with the meta-analysis conducted by Rezvani et al. [15] suggesting that aspects of the perceptual field variables (such as stimulus size, sparsity,…) play a significant role on global precedence during visual processing.

Other studies, such as resting-state fMRI studies have described large functional alterations in the salience network with healthy aging [48, 49]. In the present study, we further revealed group differences in occipital activation in the most complex conditions between the two age groups. This finding is consistent with several studies such as Cliff et al. [21] which also described age-related modulation of load-dependent neural activation in the visual cortex with increasing processing demands. Two alternative explanations can be then raised: first, reduced activation in the occipital cortex with higher attentional demands with older age may be related to altered top-down mechanism, such as inhibitory control from the frontal lobe, through altered structural and functional connectivity [50, 51]. Such hypothesis has been supported by previous studies from resting-state fMRI and electroencephalography [52] suggesting alteration of the frontal regions with top-down consequences on the visual cortex. As we did not observe activation of the frontal regions in the older group in the condition of larger visual competition, we may interpret such results as less efficient top-down mechanisms [53], which led to significantly more difficulties to ignore visual distractors during local target detection, leading to slower reaction times. Alternatively, our finding may also support the idea of an alteration of the bottom-up mechanism through reduced early sensory processing with healthy aging, in particular for high spatial frequencies that convey local information [54], which may lead less efficient local processing with increasing numbers of distractors, and therefore to reduced communication with the salience network. However, this possibility is less likely because all participants (young and older adults) presented a high level of accuracy, in all experimental conditions.

While this study has many strengths, including being among the first one investigating the neural mechanisms of local and global processing during a visual search task in older populations, we must acknowledge some limitations. Our analyses were based on cross-sectional samples and not longitudinal data. Investigating the rate of within-subject change in visual processing will be essential to understand and identify brain integrity preservation versus compensatory mechanisms in older adults [55]. Second, it will be important to test the reproducibility of our findings in a larger independent sample while also using other tasks involving more natural stimuli situations or with more realistic visual stimuli [15, 29], such as global scenes composed of several local objects (even if recent studies evidenced a link between hierarchical stimuli processes and real objects processes [56]). We also chose to use a block- rather than an event-related, design to improve our statistical power (and reduce the length of the task). However, many fMRI studies that have used an event-related design reported more subtle patterns of activations, particularly in frontal regions, between incongruent (as our target-present trials) and congruent trials [29, 37, 57], which was impossible to test in our design. Also, because of our choice of task design (e.g., block design, duration of visual stimuli presentation varying across individuals and conditions), the number of trials differed across conditions and individuals, with the younger treating the visual stimuli faster and, therefore, were seeing more stimuli than the older participants. Designing an fMRI task with constant visual presentations of stimuli across all individuals would bring complementary information on the brain mechanisms behind local-global visual processing although such design could lead to either a large drop in accuracy in older adults (in case of short visual stimuli presentation) or higher boredom (and possibly less efficient visual treatment; in case of long visual stimuli presentation) in younger adults. Third, it is also likely that further differences between subgroups within the older group could be revealed (e.g., between participants aged 50–70 years-old and participants above 70). Indeed, previous aging-related studies that reported a clear bias towards global processing in older adults had an average age of 70 years or younger [9, 11–14] while studies that revealed a bias towards local analysis were based on studies including octogenarian individuals [58]. Our sample size was too small to statistically test such differences, but such subgroups will help identify and improve our understanding of the neural correlates underlying visual processing in late adulthood. Lastly, we did not reveal any strong association between brain activation and behavioral performance. One possible explanation is our choice of ROIs that may not have been appropriate for our design. In fact, we chose to extract them from the study by Liddell et al. [26], however, the task was not exactly similar to ours and therefore, the ROIs may not have been the most relevant for us (e.g., the distance of the ROI centered on the anterior insula between [26] and our insula peak was approximately 10mm).

## Conclusion

The current study investigated the neural correlates underlying local and global processing during a visual search task in early and late adulthood. Search for a local target was less efficient as the number of visual distractors increased, as revealed by slower search times and increased recruitment of the salience network. On the other hand, this was not the case during global processing of the stimuli. Thus, the current results support the view of a local disadvantage in a context of competition (i.e., when the number of distractors increases in the visual environment), rather than an absolute global bias variation, with increased recruitment of the insula needed to detect and resist to salient information during the local condition. Importantly, older participants showed reduced activation in both the occipital lobe and insula, possibly due to variation of top-down effect from the insula that are less efficient with age, leading to a reduced activity of primary visual areas during the most complex conditions. These findings suggest that even if the same stimulus is presented, variations in brain functioning due to age are present as soon as primary visual areas, depending on which information (local or global) has to be processed. The current results support the view that even if global information processing seems preserved in older adults, top-down reduction effects on primary visual areas may lead to specific difficulties to process local visual details in daily life situations.

## Supporting information

S1 File (DOCX)
