# Supplementary material for: Neural correlates underlying local and global processing during visual search across adulthood
Source: PLoS One. 2024 Jun 21;19(6):e0303796. doi: 10.1371/journal.pone.0303796 (PMC11192325; doi:10.1371/journal.pone.0303796)

**Supporting Information**

| **S1 Table: Brain activations for the main contrasts** | | | | | |
| --- | --- | --- | --- | --- | --- |
| **Region** | **Number of voxels** | **T** | **x** | **y** | **z** |
| **All participants:** | | | | | |
| **Local > Global Contrast** | | | | | |
| R inferior Occipital | 293 | 7.61 | 30 | -93 | -6 |
| R middle Occipital | | 5.17 | 36 | -81 | 6 |
| R inferior Occipital | | 4.90 | 45 | -69 | -12 |
| L inferior Occipital | 517 | 6.71 | -27 | -96 | -9 |
| L inferior Occipital | | 6.14 | -36 | -90 | -9 |
| L inferior Occipital | | 5.19 | -42 | -66 | -9 |
| R anterior insula | 207 | 5.30 | 36 | 21 | -6 |
| R inferior Frontal, *pars triangularis* | | 3.80 | 42 | 36 | 6 |
| R middle Frontal | | 3.55 | 48 | 42 | 18 |
| R inferior Frontal, *pars opercularis* | 128 | 4.98 | 45 | 12 | 27 |
| R inferior Frontal, *pars opercularis* | | 3.51 | 54 | 15 | 39 |
| R Anterior Cingulate Cortex | 89 | 4.60 | 12 | 27 | 27 |
| R Supplementary Motor Area | | 3.74 | 6 | 15 | 51 |
| R Middle Cingulate Cortex | | 3.57 | 9 | 21 | 45 |
| L Cerebellum | 88 | 4.28 | -6 | -75 | -39 |
| L Cerebellum | | 4.09 | -6 | -72 | -21 |
| R superior Occipital | 110 | 4.11 | 24 | -75 | 48 |
| R superior Occipital | | 3.75 | 27 | -66 | 36 |
|  | | | | | |
| **Global > Local Contrast** | | |  |  |  |
| L precentral | 87 | 4.26 | -39 | -24 | 45 |
| L postcentral | | 4.15 | -33 | -27 | 51 |
|  | | | | | |
| **5D > 0D** | | | | | |
| L lingual | 1313 | 6.19 | -18 | -84 | -9 |
| R Cuneus |  | 5.10 | 15 | -93 | 9 |
| R middle Occipital | | 5.09 | 33 | -81 | 9 |
| R anterior insula | 86 | 4.71 | 39 | 21 | -6 |
| R lingual | 58 | 4.06 | 18 | -87 | -6 |
| R inferior Occipital | | 3.70 | 39 | -84 | -12 |
| R fusiform | | 3.56 | 33 | -69 | -9 |
|  | | | | | |
| **0D > 5D:** N.A. | | | | | |
|  | | | | | |
| **Younger Group:** | | | | | |
| **Local 5D > Local 0D** | | | | | |
| R Cuneus | 732 | 5.13 | 12 | -93 | 9 |
| L Lingual |  | 4.94 | -15 | -84 | -9 |
| R superior Occipital | | 4.65 | 18 | -90 | 30 |
| L middle Occipital | 223 | 4.61 | -30 | -81 | 18 |
| L Calcarine | | 3.88 | -15 | -75 | 12 |
| L middle Occipital | | 3.77 | -33 | -90 | 9 |
| R superior Occipital | 184 | 4.41 | 24 | -66 | 39 |
| R middle Occipital | | 4.38 | 36 | -81 | 9 |
| R anterior insula | 54 | 4.12 | 39 | 21 | -6 |
|  | | | | | |
| **Global 5D > Global 0D** | | | | | |
| L Lingual | 1580 | 7.91 | -15 | -84 | -6 |
| R middle Occipital | | 5.44 | 33 | -84 | 12 |
| R Cuneus |  | 5.21 | 15 | -93 | 9 |
|  | | | | | |
| **Older Group:** | | | | | |
| **Local 5D > Local 0D:** N.A. | | | | | |
|  | | | | | |
| **Global 5D > Global 0D**: N.A. | | | | | |
|  | | | | | |
| **Between-Group Differences:** | | | | | |
| **Local Conditions: Younger > Older** | | | | | |
| L Calcarine | 1209 | 7.94 | -12 | -66 | 9 |
| R Calcarine | | 7.28 | 3 | -72 | 18 |
| L Calcarine | | 6.96 | -9 | -78 | 12 |
|  | | | | | |
| **Local-5D: Younger > Older** | | | | | |
| L Calcarine | 1213 | 6.90 | -12 | -66 | 9 |
| R Calcarine | | 6.65 | 3 | -72 | 15 |
| L Calcarine | | 6.00 | -12 | -78 | 12 |
|  | | | | | |
| **Local-0D: Younger > Older** | | | | | |
| L Calcarine | 284 | 5.31 | -9 | -66 | 9 |
| L Calcarine | | 4.83 | -9 | -81 | 12 |
| L Calcarine | | 4.62 | 0 | -72 | 18 |
|  | | | | | |
| **Global Conditions: Younger > Older** | | | | | |
| L Calcarine | 446 | 5.48 | -9 | -66 | 9 |
| L Calcarine | | 5.38 | 3 | -72 | 15 |
| L Calcarine | | 5.18 | -9 | -81 | 12 |
|  | | | | | |
| **Global-5D: Younger > Older** | | | | | |
| L Calcarine | 590 | 5.38 | -9 | -66 | 9 |
| R Calcarine | | 5.28 | 3 | -72 | 15 |
| L Calcarine | | 5.27 | -9 | -69 | 18 |
|  | | | | | |
| **Global-0D: Younger > Older** | | | | | |
| N.A | | | | | |
| Coordinates are in MNI Space. Older > Younger Contrasts did not result in any significant clusters. | | | | | |

**S2 Table:** **Correlations between each ROI’s BOLD signal and in-scanner performance in each age group.**

|  |  | **L IPL** | **L IFG** | **L MFG** | **R IPL** | **R Insula** | **ACC** | **R MFG** | **R IFG** |
| --- | --- | --- | --- | --- | --- | --- | --- | --- | --- |
| **G-0D** | **Younger** | r=-0.28, p=0.087 | r=-0.29, p=0.079 | r=-0.19, p=0.262 | r=-0.09, p=0.591 | r=-0.01, p=0.933 | r=-0.15, p=0.366 | r=-0.03, p=0.864 | r=-0.07, p=0.678 |
|  | **Older** | r=0.11, p=0.521 | r=0.06, p=0.726 | r=0.02, p=0.888 | r=0.06, p=0.718 | r=0, p=0.991 | r=-0.01, p=0.972 | r=-0.14, p=0.436 | r=-0.08, p=0.663 |
| **G-5D** | **Younger** | r=0.29, p=0.08 | **r=0.37, p=0.022** | r=0.31, p=0.057 | r=0.22, p=0.192 | **r=0.35, p=0.033** | **r=0.47, p=0.003** | r=0.24, p=0.15 | **r=0.45, p=0.005** |
|  | **Older** | r=0.03, p=0.886 | r=-0.01, p=0.972 | r=-0.14, p=0.406 | r=-0.08, p=0.662 | r=-0.24, p=0.16 | r=-0.09, p=0.609 | r=-0.14, p=0.438 | r=-0.27, p=0.115 |
| **L-0D** | **Younger** | r=-0.09, p=0.611 | r=-0.09, p=0.583 | r=-0.09, p=0.597 | r=-0.21, p=0.212 | r=-0.08, p=0.63 | r=-0.22, p=0.183 | r=-0.15, p=0.354 | r=-0.25, p=0.131 |
|  | **Older** | r=-0.18, p=0.314 | r=-0.13, p=0.458 | r=-0.15, p=0.375 | r=-0.3, p=0.076 | r=-0.1, p=0.554 | r=-0.28, p=0.109 | **r=-0.57, p=2E-4** | **r=-0.46, p=0.006** |
| **L-5D** | **Younger** | r=-0.01, p=0.943 | r=-0.28, p=0.093 | r=-0.09, p=0.586 | r=-0.06, p=0.735 | r=-0.26, p=0.118 | r=-0.11, p=0.526 | r=-0.02, p=0.9 | r=-0.05, p=0.769 |
|  | **Older** | **r=0.38, p=0.025** | **r=0.38, p=0.024** | **r=0.37, p=0.03** | r=0.24, p=0.17 | r=0.10, p=0.58 | **r=0.49, p=0.003** | r=0.17, p=0.33 | r=0.15, p=0.40 |
| Bold values show significant at p<0.05, uncorrected for multiple comparison. Spearman Correlations were conducted. | | | | | | | | | |

**S1 Figure. Eight Regions of Interest selected based on Liddell et al. [26].**


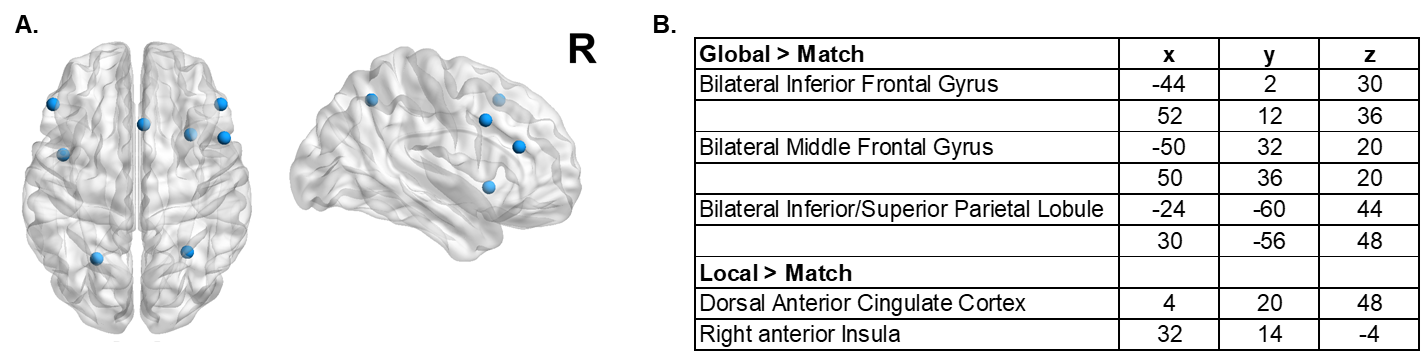

Supplement: S1 File — (DOCX) [file pone.0303796.s001.docx]
